# N-acetylcysteine inhibit biofilms produced by *Pseudomonas aeruginosa*

**DOI:** 10.1186/1471-2180-10-140

**Published:** 2010-05-12

**Authors:** Tiemei Zhao, Youning Liu

**Affiliations:** 1 Department of Respiratory diseases, Chinese PLA General Hospital, Beijing, China

## Abstract

**Background:**

*Pseudomonas aeruginosa *is a common pathogen in chronic respiratory tract infections. It typically makes a biofilm, which makes treatment of these infections difficult. In this study, we investigated the inhibitory effects of N-acetylcysteine (NAC) on biofilms produced by *P. aeruginosa*.

**Results:**

We found that minimum inhibitory concentrations (MICs) of NAC for most isolates of *P. aeruginosa *were 10 to 40 mg/ml, the combination of NAC and ciprofloxacin (CIP) demonstrated either synergy (50%) or no interaction (50%) against the *P. aeruginosa *strains. NAC at 0.5 mg/ml could detach mature *P. aeruginosa *biofilms. Disruption was proportional to NAC concentrations, and biofilms were completely disrupted at 10 mg/ml NAC. Analysis using COMSTAT software also showed that PAO1 biofilm biomass decreased and its heterogeneity increased as NAC concentration increased. NAC and ciprofloxacin showed significant killing of *P. aeruginosa *in biofilms at 2.5 mg/ml and > 2 MIC, respectively (*p *< 0.01). NAC-ciprofloxacin combinations consistently decreased viable biofilm-associated bacteria relative to the control; this combination was synergistic at NAC of 0.5 mg/ml and CIP at 1/2MIC (*p* < 0.01).  Extracellular polysaccharides (EPS) production by *P. aeruginosa *also decreased by 27.64% and 44.59% at NAC concentrations of 0.5 mg/ml and 1 mg/ml.

**Conclusions:**

NAC has anti-bacterial properties against *P. aeruginosa *and may detach *P. aeruginosa *biofilms. Use of NAC may be a new strategy for the treatment of biofilm-associated chronic respiratory infections due to *P. aeruginosa*, although it would be appropriate to conduct clinical studies to confirm this.

## Background

*Pseudomonas aeruginosa *is a known common pathogen in respiratory tract infections. These diseases are usually chronic, such as pulmonary infections in intubated patients and for patients with cystic fibrosis (CF), bronchiectasis, diffuse panbronchiolitis [1,2] and chronic obstructive pulmonary disease (COPD). One reason why treating these infections is difficult is the production of biofilms by *P. aeruginosa *[3]. Organisms in the biofilm become more resistant than planktonic bacteria to physical and chemical attacks, such as by chemotherapeutic reagents. Discovering substances that inhibit biofilm formation and/or disrupt established biofilms is essential for treating these diseases.

N-acetylcysteine (NAC) is a mucolytic agent that has anti-bacterial properties. NAC also decreases biofilm formation by a variety of bacteria [4-6] and reduces the production of an extracellular polysaccharide matrix, while promoting the disruption of mature biofilms [4,7]. The effect of NAC on *P. aeruginosa *biofilms has not been extensively studied, and a better understanding of bacterial responses to NAC may facilitate its use as a biofilm inhibitor. Thus, we investigated the effects of NAC for (i) anti-bacterial properties, (ii) detachment of biofilms, (iii) viable cells in biofilms and (iv) production of extracellular polysaccharides (EPS) by *P. aeruginosa*.

## Results

### Susceptibility of *P. aeruginosa *strains to NAC and the in vitro interactive effects of NAC and ciprofloxacin

Twenty *P. aeruginosa *strains were isolated from respiratory samples. The minimum inhibitory concentrations (MICs) of NAC for 18 *P. aeruginosa *isolates were 10 to 40 mg/ml, and MICs for another 2 isolates were > 40 mg/ml. The combination of NAC and ciprofloxacin demonstrated either synergy (50%) or no interaction (50%) against the *P. aeruginosa *strains; antagonism was not observed.

### Interpretations of biofilm production

Using the criteria of Stepanovic et al, *P. aeruginosa *strains were divided into the following categories: 3 (15%) were weak biofilm producers; 10 (50%) were moderate biofilm producers; 7 (35%) were strong biofilm producers.

### Effects of NAC on biofilms of *P. aeruginosa *PAO1 and quantitative analysis using COMSTAT software

**Figure 1:**
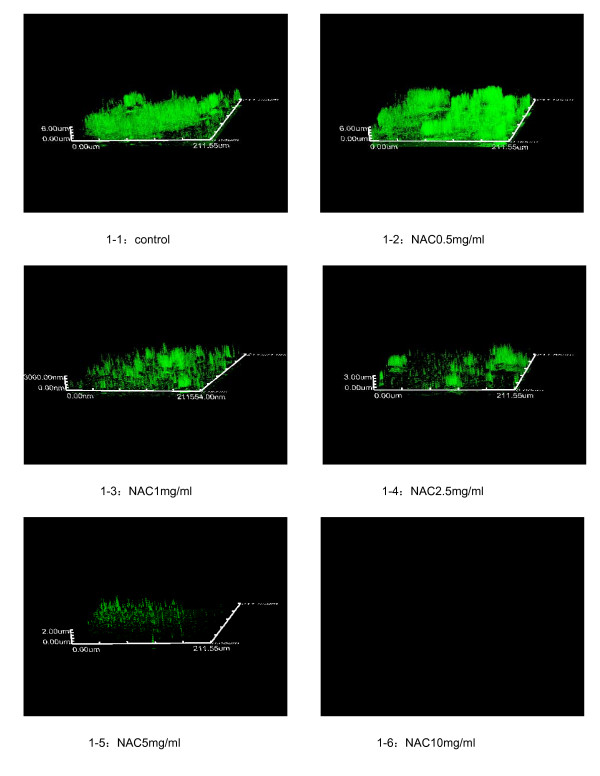
**Biofilms of P. aeruginosa PAO1 expressing a GFP plasmid (pMRP9-1) exposed to NAC (1-1, 1-2, 1-3, 1-4, 1-5, 1-6 showed different concentrations)**. CLSM was used to create three-dimensional reconstructions of the PAO1 biofilms. Each side of image was 210 μm.

**Figure 2:**
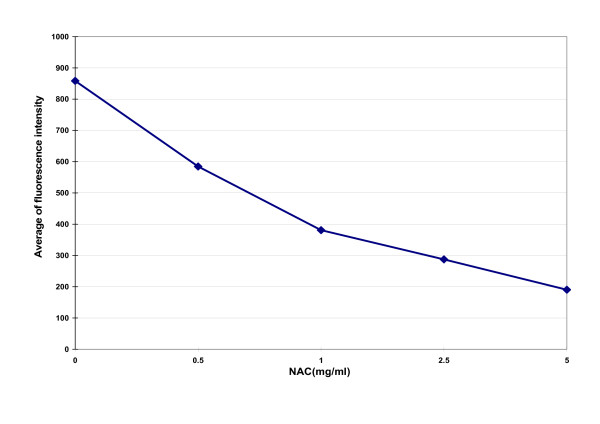
**Fluorescence intensity in each fixed CLSM scanning area after treatment with NAC**. NAC at 1 mg/ml, 2.5 mg/ml and 5 mg/ml significantly decreased the fluorescence of PAO1 biofilms after 24 hours exposure compared with control (*P *< 0.01).

As shown in Figure 1, biofilms were observed using confocal laser scanning microscopy (CLSM) and three-dimensional images were reconstructed by Olympus FV10-ASM1.7 Software. A GFP-plasmid was inserted into PAO1, which allowed the detection of live bacteria by fluorescence. Observed by CLSM, PAO1 grew in a characteristic pattern with a lawn of bacterial growth on the surface. These results showed that NAC disrupted and inhibited PAO1 biofilms, fluorescence and thickness decreased after exposure to NAC, and there was an NAC dose-dependent effect. Almost no fluorescence was detected after 10 mg/ml NAC treatment, indicating that very few to no live PAO1 were present. Decreased GFP detection levels were associated with increasing concentrations of NAC in each fixed scanning area (Figure 2).

When analyzed using COMSTAT software, *P. aeruginosa *biofilms showed significant structural differences in the presence of the NAC regimen (Table 1). The biomass, substratum coverage, average thickness, maximum thickness and surface area of the biomass all decreased for biofilms grown in the presence of NAC. The surface to volume ratio and roughness coefficients showed the opposite trends.

**Table 1:** Effects of NAC (mg/ml) on biofilm structures of PAO1

Features	control	NAC 0.5	NAC 1	NAC 2.5	NAC 5
Biomass (μm^3^/μm^2^)	2.79 ± 0.64	1.63* ± 0.46	0.98* ± 0.57	0.34* ± 0.17	0.23* ± 0.12
Substratum coverage	0.52 ± 0.19	0.34 ± 0.11	0.35 ± 0.19	0.20* ± 0.08	0.21* ± 0.11
Average thickness (μm)	2.70 ± 0.80	1.47* ± 0.47	0.75* ± 0.51	0.19* ± 0.16	0.01* ± 0.01
Maximum thickness (μm)	10.20 ± 1.64	8.40* ± 1.92	5.20* ± 1.64	3.00* ± 0.80	1.60* ± 0.48
Surface area of biomass (μm^2^)	162515.9 ± 27990.3	99499.0* ± 25130.4	102665.0* ± 50400.6	49869.1* ± 24393.6	41504.3* ± 18129.7
Surface to volume ratio (μm^2^/μm^3^)	1.39 ± 0.33	1.41 ± 0.12	2.66* ± 0.56	3.64* ± 0.78	4.47* ± 0.66
Roughness coefficient	1.12 ± 0.19	1.43 ± 0.14	1.53* ± 0.27	1.72* ± 0.25	1.97* ± 0.02

Note: n = 10 image stacks, *compared with control, *P *< 0.01

### Viable cell counts after treatment with NAC combined with CIP

Results for viable cell counts in biofilms are shown in Table 2. NAC had an independent anti-microbial effect on biofilm-associated *P. aeruginosa *at 2.5 mg/ml (p < 0.01). Compared with the control, there were significant differences at ciprofloxacin (CIP) of 2 MIC, 4 MIC or 8 MIC (p < 0.01). NAC-ciprofloxacin combinations consistently decreased viable biofilm-associated bacterial counts relative to the control. This combination was synergistic at NAC of 0.5 mg/ml and CIP of 1/2MIC (p < 0.01).

**Table 2:** Viable counts of *P. aeruginosa *biofilm bacteria treated with NAC combined with ciprofloxacin (lg [CFU/cm^2^])

NAC (mg/ml)	ciprofloxacin (MIC)					
	
	0	1/2	1	2	4	8
0	7.11 ± 0.34	6.96 ± 0.34	6.95 ± 0.31	6.84 ± 0.32	6.76 ± 0.29	6.60 ± 0.30
0.5	6.97 ± 0.31	6.70 ± 0.31	6.65* ± 0.33	6.40* ± 0.46	6.37* ± 0.33	6.06* ± 0.48
1	6.87 ± 0.34	6.58* ± 0.26	6.47* ± 0.33	6.23* ± 0.37	5.94* ± 0.56	5.62* ± 0.59
2.5	6.45* ± 0.27	6.22* ± 0.25	6.15* ± 0.26	6.03* ± 0.35	5.76* ± 0.58	5.05* ± 0.35

Note: n = 20 strains, *compared with NAC at 0 mg/ml and the same concentration of ciprofloxacin, *P *< 0.01

### Effect of NAC on extracellular polysaccharides (EPS) production

EPS production by *P. aeruginosa *decreased significantly in the presence of NAC. The amount of EPS produced by *P. aeruginosa *decreased, on average, by as much as 27.46% and 44.59% in the presence of 0.5 mg/ml and 1 mg/ml NAC, respectively.

## Discussion

NAC is considered a non-antibiotic drug that has anti-bacterial properties. In 1977, Parry and Neu [8] found that NAC had the characteristics to inhibit the growth of both gram-positive and gram-negative bacteria, including *S. aureus, P. aeruginosa, K. pneumoniae *and *Enterobacter clocae. P. aeruginosa *was more susceptible than most of the other tested microorganisms (MIC 2-20 μg/ml), *P. aeruginosa *strains were inhibited synergistically by NAC and carbenicillin or ticarcillin. Roberts and Cole [9] found that 2%-5% of NAC was anti-bactericidal against *P. aeruginosa*, the effect of the carbenicillin on *P. aeruginosa *was augmented by low concentrations of NAC, and the MIC of the organisms to carbenicillin was reduced from 16 μg/ml to 1 μg/ml in the presence of one per cent NAC. The mechanism for the anti-bacterial effect of NAC may be that it acts by competitively inhibiting amino acid (cysteine) utilization or, by virtue of possessing a sulfhydryl group, may react with bacterial cell proteins. Our results are consistent with those of Roberts and Cole, as most of the *P. aeruginosa *strains were inhibited at concentrations < 40 mg/ml of NAC, and a higher percentage of synergistic combinations with NAC was observed with ciprofloxacin (50%). This means that NAC and ciprofloxacin may be used together to treat *P. aeruginosa *infections.

Due to its ability to produce a biofilm, *P. aeruginosa *is responsible for some chronic pulmonary infections, such as those in cystic fibrosis (CF), bronchiectasis and chronic obstructive pulmonary disease (COPD). It also implicates that the infections are associated with endotracheal tubes [10,11]. *P. aeruginosa *eventually causes infections in most patients with CF, and once a chronic infection is established, eradication of *P. aeruginosa *strains is nearly impossible. Patients with bronchiectasis who are colonized by *P. aeruginosa *exhibit more advanced diseases and more severe impairments of pulmonary function compared with those who remain free of colonization [12-14]. Some observations suggest that *P. aeruginosa *is a more common cause of infection as COPD advances [15,16]. Recently, Martínez-Solano et al. [17] showed that patients with COPD were usually infected with at least 1 *P. aeruginosa *clone that remained in the lungs for years. During chronic infection, each clone diversified, and isolated from samples of infected lungs tended to produce more biofilm compared with the isolation from blood samples. The structure and physiological characteristics of a biofilm confer an inherent resistance to anti-microbial agents. The MICs of anti-microbial agents can be increased 100- to 1000-fold when bacteria grow in biofilms as compared to planktonic bacteria [18]. Therapy almost always fails to eradicate the bacteria in biofilms. Therefore, it is important to search for an effective drug to eradicate the biofilm.

Previous studies have shown that NAC could decrease biofilm formation by a variety of bacteria [4-6] and that it inhibited bacterial adherence, reduced the production of extracellular polysaccharide matrix, while promoting the disruption of mature biofilms, and reduced sessile cell viability [4,7]. Olofsson [7] studied the biofilms of 10 bacterial strains isolated from a paper mill. These results showed that EPS production decreased significantly in the presence of NAC (0.25 mg/ml). Although the growth didn't affected the most of tested bacteria, the average reduction in the amount of EPS produced was 58% ± 20%; the presence of NAC reduced the number of attached multi-species community bacteria by as much as 76% ± 46%. There is only one article demonstrated the inhibitory effect of NAC on *P. aeruginosa *adherence and biofilm formation in vitro by the number of viable cell counts previously, and also revealed that ciprofloxacin/NAC combination showed the highest ability to inhibit biofilm synthesis and disrupt preformed mature biofilms [19]. In our research, inhibitory effects of drugs on biofilms not only determined by the viable count technique, but also were imaged using CLSM and quantified biofilm structures by COMSTAT program, EPS production in the presence of NAC also be examined quantitatively. CLSM can provide three-dimensional, noninvasive inspection and computer reconstruction of mature biofilms without appreciable distortion of architecture in a manner similar to computer-assisted tomography and magnetic resonance imaging methods. COMSTAT comprises some features for quantifying three-dimensional biofilm image stacks [20]. Biomass represents the overall volume of the biofilm, substratum coverage reflects how efficiently the substratum is colonized by bacteria of the population, the surface area of biomass is the area which summation of all biomass voxel surfaces exposed to the background, the surface to volume ratio is the surface area divided by the bio-volume which indicates how the biofilm adapts to the environment, roughness provides a measure of how much the thickness of the biofilm varies, and it is also an indicator of biofilm heterogeneity. Our results showed that NAC dispersed the biofilms formed by *P. aeruginosa*. By visual inspection of CLSM images, NAC disrupted and inhibited PAO1 biofilms, fluorescence and thickness decreased after exposure to NAC and there were dose-dependent effects. Biofilms were nearly detached at 10 mg/ml NAC. Using COMSTAT software, the PAO1 biofilm biomass decreased and its heterogeneity increased gradually in direct proportion to the NAC concentration. NAC also had an independent anti-microbial effect on biofilm-associated *P. aeruginosa *at 2.5 mg/ml (*P *<	0.01) and had a synergistic effect with CIP. Bacterial exopolysaccharides are the main component of the biofilm, which creates an efficient scavenging system for trapping and concentrating essential minerals and nutrients from the surrounding environment. Our research showed that the amounts of EPS produced by *P. aeruginosa *strains were also significantly inhibited by 0.5 and 1 mg/ml of NAC. Taking into account the results given above, NAC may be a potent agent for treating *P. aeruginosa *biofilms associated infections, and can be used in combination with ciprofloxacin.

Stafanger [21] studied the effect of peroral NAC in patients with cystic fibrosis and chronic pulmonary *P. aeruginosa *infection, a significant improvement of the spirometric values was proved after NAC treatment in the patients with peak expiratory flow rate below or equal to 70% of predicted normal values. Stey [22] reviewed the publications on the effect of oral NAC in chronic bronchitis, eleven randomized controlled NAC trials were analysed (a total of 2,011 patients), concluded that oral NAC reduced the risk of exacerbation and improved symptoms in patients with chronic bronchitis compared with palcebo. But the benefit it achieved still remains unclear. We are not sure whether it took into account the other elements such as anti-bacterial activities and detach biofilms or not? It needs further study.

NAC can be administered by nebulization or direct instillation, orally or intravenously. The concentrations tested in our study are much higher than those reach in serum when administer by an intravenous or oral route. Nevertheless, it may be possible that using local respiratory application (10% solution may be used undiluted for inhalation) obtains useful concentrations to disrupt biofilms and control biofilm-associated infections of *P. aeruginosa*.

## Conclusions

In conclusion, our results suggest that NAC has anti-bacterial properties against *P. aeruginosa *and may detach *P. aeruginosa *biofilms. It may be a new strategy for the treatment of biofilm-associated chronic respiratory infections, although it would be appropriate to conduct in vivo animal models and clinical studies to confirm this.

## Methods

### Bacterial strains

*P. aeruginosa *PAO1 expressing a green fluorescent protein (GFP) plasmid (pMRP9-1) was kindly donated by Dr. E. P. Greenberg (University of Washington, Seattle). An additional 20 strains of *P. aeruginosa *isolated from respiratory samples were studied.

### Determination of minimum inhibitory concentrations (MIC) and drug-drug interactions

Crystalline NAC (Sigma-Aldrich, USA) was dissolved in distilled water to make a 100 mg/ml solution; the pH of solution was adjusted to 7.2 before use. Stock solution of ciprofloxacin (National Institute for the Control of Pharmaceutical and Biological Products, China) was prepared at concentrations of 4096 μg/ml in the distilled water.

MICs of NAC and ciprofloxacin were determined using a broth micro-dilution assay according to Clinical Laboratory Standards Institute (CLSI) guidelines [23]. Each well of a 96-well microtiter plate containing 100 μl from a series of diluted NAC with Mueller-Hintor broth was inoculated with 100 μl of *P. aeruginosa *suspension (6-hour broth cultures); the final inoculum was 5 × 10^5 ^CFU/ml. After 20 h incubation in air at 35°C, the wells were inspected for microbial growth and the MIC was defined as the lowest concentration that inhibited the growth of bacteria. Positive (bacterial suspension) and negative (broth) controls were also included.

In vitro antibacterial activities of ciprofloxacin in combination with NAC were determined by chequerboard MIC assay as previously described [24]. Mueller-Hinton broth was used. Seven doubling dilutions of NAC and 11 doubling dilutions of ciprofloxacin were tested. After drug dilution, microbroth dilution plates were inoculated with each organism to yield the appropriate density (10^5 ^CFU/ml) in a 100 μl final volume and incubated for 20 h at 35°C in ambient air. The fractional inhibitory concentration index (FICI) was calculated for each combination using the following formula: FICA + FICB = FICI, where FICA = MIC of drug A in combination/MIC of drug A alone, and FICB = MIC of drug B in combination/MIC of drug B alone. The FICI was interpreted as follows: synergy = FICI ≤ 0.5; no interaction = FICI >0.5-≤ 4; antagonism = FICI > 4.

### Interpretation of biofilm production

Biofilm production was determined using a spectrophotometric method described by Stepanovic et al [25]. Briefly, stationary-phase 18-h cultures of *P. aeruginosa *were diluted with fresh trypticase soy broth (TSB), and standardized to contain 1 × 10^6 ^CFU/ml. Aliquots (0.2 ml) of the diluted cultures were added to 96-well sterile flat-bottom polystyrene tissue culture plates (Costar, USA). After 24 h incubation at 37°C, the contents of the tissue culture plates were gently aspirated, then washed 3 times with sterile PBS (pH 7.2). Slime and adherent organisms were fixed by 200 μl of 99% methanol for 20 min, stained with 200 μl crystal violet (1%) for 20 min. Excess stain was removed by placing the plates under running distilled water, and then the plates were air dried. The dye bound to the cells was resolubilized with 160 μl of 95% ethanol. The optical density of the stained adherent films was read with a microplate Reader (Pulang New Technology Corporation, China) at a wavelength of 570 nm. Measurements were performed in triplicate and repeated 3 times. Interpretation of biofilm production was according to the criteria of Stepanovic et al [25] (Table 3).

**Table 3:** Criteria of interpretation of biofilm production

Biofilm production	average optical density (OD)
no biofilm producer	≤ ODc
weak biofilm producer	ODc < ~ ≤ 2 × ODc
moderate biofilm producer	2 × ODc < ~ ≤ 4 × ODc
strong biofilm producer	> 4 × ODc

Note: optical density cut-off value (ODc) = average OD of negative control + 3 × SD of negative control.

### PAO1 biofilm analysis using CLSM

TSB (4 ml) was dispensed in a culture dish containing a sterile cover slip (MatTek, USA). Then, 50 μl of a bacterial suspension (1.5 × 10^8 ^CFU/ml) was inoculated into the dish and incubated aerobically at 37°C for 6 days. The biofilms deposited on cover slips were gently rinsed with sterile PBS to remove bacterial cells, except for those included in the biofilm. After rinsing, the biofilm was soaked in a diluent containing NAC (0, 0.5, 1, 2.5, 5, 10 mg/ml) for 24 h at 37°C. After rinsing with PBS, the samples were examined for the degree of biofilm removal by observation under a confocal laser scanning microscopy (CLSM). To analyze the effects of NAC on biofilms, 2 independent biofilm experiments were performed. From each cover slip, 5 image stacks were acquired at different positions; thus, 10 image stacks were analyzed for each concentration of NAC. Images were acquired at 1 μm intervals down through the biofilm and, therefore, the number of images in each stack varied according to the thickness of the biofilm. All microscopic observations and image acquisitions used CLSM (Olympus FV1000, Japan). Images were obtained with a 60× objective lens and laser excitation at 488 nm. Z-series of optical sections were reconstructed into three-dimensional images by Olympus FV10-ASM 1.7 Software. Fluorescence intensity in each fixed scanning area was measured.

The biofilm structure was quantified from the confocal stacks using the image analysis software package COMSTAT (kindly donated by A. Heydorn, Technical University of Denmark, Lyngby) [20]. This software can interface with Matlab and utilizes Matlab's image analysis software toolbox. COMSTAT offers an array of functions and is capable of generating up to 10 different statistical parameters for quantifying the 3-dimensional biofilm structure. For this study, 7 COMSTAT parameters were used to determine the differences between biofilms grown under each of the 5 NAC concentrations. These parameters were biomass, substratum coverage, maximum thickness, average thickness, surface area of biomass, surface to volume ratio and roughness coefficient.

### Detection of viable cells in biofilms using MTT assay

Dimethylthiazol diphenyltetrazolium bromide (MTT) and extraction buffer were prepared as previously described [26]. In brief, MTT was dissolved at a concentration of 5 mg/ml in PBS. Extraction buffer was prepared by dissolving 20% (wt/vol) sodium dodecyl sulfate (SDS) at 37°C in a solution of 50% each of *N,N*-dimethylformamide (DMF) and demineralized water; the pH was adjusted to 4.7.

MTT assay. Twenty μl of the 5-mg/ml MTT stock solution was added to each well of a 96-well microtiter plate (Costar, USA) containing 190 μl of bacteria. After incubation for 2 h at 37°C, 90 μl of extraction buffer was added to each well. After thorough extraction, optical densities were measured at 595 nm using a microplate reader (Pulang New Technology Corporation, China).  MHB (incubated with MTT and extraction buffer) was used as a blank control. The assay was calibrated using series dilutions of *P. aeruginosa *ATCC 27853 as standards, which had been subjected to the same procedure.

TSB (2 ml) was dispensed in a 24-well microplate well (Costar, USA) with a cut silicone sheet as a matrix for the bacterial biofilm. Then, 50 μl of a bacterial suspension (1.5 × 10^8 ^CFU/ml) was inoculated into the well and incubated aerobically at 37°C for 6 days. The biofilm deposited on a silicone sheet was gently rinsed with sterile saline to remove bacterial cells, except for those included in the biofilm. After rinsing, the biofilm was soaked in 1 of the following treatment solutions for 24 h at 37°C: NAC (0, 0.5 mg/ml, 1 mg/ml, 2.5 mg/ml) and ciprofloxacin (0, 1/2MIC, 1MIC, 2MIC, 4MIC, 8MIC) combinations according to a checkerboard design. Then, the sheets were rinsed 3 times with PBS to remove planktonic bacteria, individually sonicated for 10 min and vortexed for 3 min in 1 ml of MHB. The number of viable cells was counted by the method described above. The lg (CFU) per cubic centimeter values of sheets for different groups were calculated.

### Measurement of extracellular polysaccharides (EPS)

The amount of carbohydrates produced by each bacterial strain was determined using a modified version of the acid hydrolysis method of Dall and Herndon [27]. Briefly, polysaccharides were precipitated with ethanol and then dehydrated with concentrated acid to a furfural. When tryptophan reacted with furfural, a condensation product was formed, which developed a brownish violet color. The amounts of EPS were determined spectrophotometrically by measuring the absorbance at 490 nm.

Six-day old bacterial biofilms on silicone sheets, grown as described above, were gently rinsed with PBS. After rinsing, the biofilm was placed in alginate producing (AP) medium described by Terry et al. [28] for 48 h, then soaked in AP medium containing NAC (0, 0.5 mg/ml, 1 mg/ml) for an additional 24 h, gently rinsed with PBS to remove bacterial cells, except for those included in the biofilm, individually sonicated for 10 min and vortexed for 3 min in 1 ml of PBS. The suspension was adjusted to 90% light transmittance at 400 nm (about 2.1 × 10^7 ^CFU/ml), then centrifuged at 950 × *g *for 10 min, and the supernatant was filtered through a sterile 0.22-μm membrane filter. Then, 0.5 ml of the supernatant fluid, which contained the polysaccharide, was precipitated by adding it in drops to 4 ml of cold absolute ethanol. Polysaccharides were pelleted by centrifugation (2,400 × *g*, 15 min) and resuspended in 200 μl of distilled water, after which they were digested with 700 μl of sulfuric acid (77%) to form monosaccharides. Samples were cooled for 10 min in an ice bath, then 0.1 ml of cold tryptophan (1%, wt/wt) was added to each tube and mixed. After heating in a boiling bath for 20 min, the tubes were cooled on ice, and the absorbance at 490 nm was read with a spectrophotometer (Pulang New Technology Corporation, China) using a PBS blank subjected to the same procedure. The amount of EPS was expressed in μg/μl. The assay was calibrated using a dextran standard (Dextran T500, Pharmacia) subjected to the same procedure.

### Statistical analysis

Results were expressed as the means ± SD. One-way ANOVA was used to compare groups; multiple comparisons used the Least-significant difference (LSD) method. Analysis used SPSS 13.0 for Windows. *P*-values < 0.01 indicated significant differences.

## Authors' contributions

TZ conceived of the study and carried out the main research. YL participated in the design of the study and performed the statistical analysis. TZ and YL wrote the paper. All authors read and approved the final manuscript.
